# Performance of Aldosterone-to-renin Ratio Before Washout of Antihypertensive Drugs in Screening of Primary Aldosteronism

**DOI:** 10.1210/clinem/dgae094

**Published:** 2024-02-21

**Authors:** Xinyu Liu, Sufang Hao, Jin Bian, Ying Lou, Huimin Zhang, Haiying Wu, Jun Cai, Wenjun Ma

**Affiliations:** Hypertension Center, Fuwai Hospital, National Center for Cardiovascular Diseases, Chinese Academy of Medical Sciences and Peking Union Medical College, Beijing, 100037, China; Hypertension Center, Fuwai Hospital, National Center for Cardiovascular Diseases, Chinese Academy of Medical Sciences and Peking Union Medical College, Beijing, 100037, China; Hypertension Center, Fuwai Hospital, National Center for Cardiovascular Diseases, Chinese Academy of Medical Sciences and Peking Union Medical College, Beijing, 100037, China; Hypertension Center, Fuwai Hospital, National Center for Cardiovascular Diseases, Chinese Academy of Medical Sciences and Peking Union Medical College, Beijing, 100037, China; Hypertension Center, Fuwai Hospital, National Center for Cardiovascular Diseases, Chinese Academy of Medical Sciences and Peking Union Medical College, Beijing, 100037, China; Hypertension Center, Fuwai Hospital, National Center for Cardiovascular Diseases, Chinese Academy of Medical Sciences and Peking Union Medical College, Beijing, 100037, China; Hypertension Center, Fuwai Hospital, National Center for Cardiovascular Diseases, Chinese Academy of Medical Sciences and Peking Union Medical College, Beijing, 100037, China; Hypertension Center, Fuwai Hospital, National Center for Cardiovascular Diseases, Chinese Academy of Medical Sciences and Peking Union Medical College, Beijing, 100037, China

**Keywords:** primary aldosteronism, aldosterone-to-renin ratio, discontinuation of antihypertensive drugs, secondary hypertension

## Abstract

**Objective:**

The aim of this study is to evaluate performance of aldosterone-to-renin ratio (ARR) before washout of antihypertensive drugs as a screening test for primary aldosteronism (PA).

**Methods:**

This retrospective analysis included consecutive patients screening for secondary hypertension during a period from January 2017 to May 2022 at the authors’ institute. For inclusion in the final analysis, ARR had to be available prior to as well as after discontinuation of antihypertensives. Patients with ARR ≥2.4(ng/dL)/(μIU/mL) after washout proceeded to confirmatory tests. Diagnosis of PA was established based on a positive result of the confirmatory test. The diagnostic accuracy of ARR prior to the washout in predicting PA is shown as sensitivity, specificity, positive predictive value (PPV), and negative predictive value (NPV).

**Results:**

The analysis included a total of 1306 patients [median age of 50.2 (41.0-59.0) years, 64.0% male]. Confirmatory tests showed PA in 215(16.5%) patients and essential hypertension (EH) in the remaining 1091(83.5%) patients. In comparison to the second screening test, the first screening test (before washout of antihypertensives) yielded lower plasma aldosterone and higher renin and consequently lower ARR in both the PA and EH groups. At a cutoff of .7(ng/dL)/(μIU/mL), ARR before washout had 96.3% sensitivity, 61.2% specificity, .33 PPV, and .99 NPV. At a lower cutoff of .5(ng/dL)/(μIU/mL), the sensitivity, specificity, PPV, and NPV were 97.7%, 52.0%, .29, and .99, respectively.

**Conclusion:**

ARR prior to washout of antihypertensives is a sensitive screening test for PA. Washout of antihypertensives could be omitted and further investigation for PA is not warranted if ARR is ≤ .7(ng/dL)/(μIU/mL) before washout.

Primary aldosteronism (PA) is the most common and potentially curable form of secondary hypertension (1). The estimated prevalence of PA is .7% to 8.5% in primary care and 4.7% to 24.0% in hypertension referral centers (2-4). In comparison to age- and blood pressure-matched patients with essential hypertension (EH), patients with PA are at higher risk of cerebral and cardiovascular morbidity, renal complications, and mortality (5, 6). Early identification and prompt treatment are thus important.

Diagnosis of PA is based on recognition of characteristic elevation of aldosterone production and suppressed renin and typically involves 3 steps (ie, screening, confirmation/exclusion, and then subtyping). Plasma aldosterone-to-renin ratio (ARR) is recommended by all major guidelines as the standard screening test (7-9). Since plasma ARR is affected by most first-line antihypertensives, antihypertensive drugs are discontinued for at least 2 to 4 weeks before the screening (10-13). Discontinuation of antihypertensives in preparation for screening test, however, is associated with increased risks of a variety of complications, including hypertensive crisis, hypokalemia, atrial fibrillation, and heart failure, particularly in patients with severe hypertension (11).

We conducted a retrospective study to explore the possibility of measuring ARR without discontinuing hypertensives as a screening test for PA. We used the STARD checklist when writing our report (14). Results are reported later.

## Materials and Methods

### Study Subjects

This retrospective study included consecutive patients referred to the Hypertension Center of the National Center for Cardiovascular Diseases/Fuwai Hospital (Beijing, China) screening for secondary hypertension between January 2017 and May 2022. The study was approved by the Institutional Ethics Committee of Fuwai Hospital (approval number: 2023-2168).

For inclusion in the analysis, the patients must be taking at least 1 of the antihypertensives with substantial impact on ARR (eg, angiotensin-converting enzyme inhibitors, angiotensin-II blockers, β-adrenergic blockers, dihydropyridine calcium antagonists, mineralocorticoid receptor antagonists, and potassium-wasting diuretics). The first screening test was conducted prior to withdrawal of the aforementioned antihypertensives, and the second test was conducted after washout (4 weeks for mineralocorticoid receptor antagonists and potassium-wasting diuretics, 2 weeks for others). During the washout period, patients were treated with a-blockers and nondihydropyridine calcium channel blockers if necessary.

Cases with 1 or more of the following conditions were excluded from the analysis: (1) the use of nonsteroidal anti-inflammatory drugs, central alpha-receptor agonists, and licorice; (2) renal insufficiency (serum creatine ≥133μmol/L); (3) pregnant or lactating women. Patients with a subsequent diagnosis of secondary hypertension other than PA were also excluded from the final analysis.

### Screening and Confirmatory Tests

The screening test was conducted after the subjects had been in an upright position for at least 2 hours following the current guidelines (9, 15). Hypokalemia was corrected before the screening; sodium intake was not restricted. ARR was calculated as plasma aldosterone concentration in ng/dL divided by direct renin concentration in μIU/mL.

Patients with positive screening test (ARR ≥2.4 upon the second test, ie, after washout) (9, 16-18) proceeded to confirmatory tests, including saline infusion test and captopril challenge test. The final diagnosis of PA was established based on a positive result in at least 1 of the confirmatory tests.

For the captopril challenge test, patients received 50 mg captopril orally (25 mg if systolic pressure was <120 mmHg) at 8:00 to 9:00 Am after at least 1 hour in the upright position. Plasma renin and aldosterone were measured at baseline and 2 hours after the captopril challenge. Patients remained upright throughout the test. Plasma aldosterone concentration > 11 ng/dL at 2 hours after the captopril challenge was considered positive (8, 19). For the saline infusion test, patients received an intravenous infusion of 2L .9% saline over 4 hours in the supine position. Plasma aldosterone concentration after the infusion at >10 ng/dL was considered positive (8, 9).

Plasma concentration of renin and aldosterone was measured using chemiluminescence immunoassays (Liaison® DiaSorin, Italy). Renin concentration below the lower detection limit of .5 μIU/mL was considered as .5 μIU/mL for calculation. Intra- and interassay coefficients were <5% for both plasma renin and aldosterone.

### Statistical Analysis

Continuous variables with normal distribution (as verified by the Shapiro–Wilk test) were compared between the patients with PA vs EH using Student's t-test for independent samples and expressed as mean ± SD. Continuous variables with skewed distribution were compared using the Mann–Whitney U test and expressed as median and interquartile range. Categorical variables were compared using the χ^2^ test and expressed as number and percentage. Plasma concentration of aldosterone and renin and ARR in the first vs the second screening were compared using the Wilcoxon signed-rank test. *P* < .05 (two-sided) was considered statistically significant. All statistical analyses were conducted using SPSS 23.0 (SPSS Inc., Chicago, IL, USA).

Performance of the first screening test was examined using MedCalc 15.2 (MedCalc Software, Ostend, Belgium). The results are shown as sensitivity, specificity, positive predictive value (PPV), negative predictive value (NPV), and their 95% confidence interval (CI). The DeLong test was used to compare the area under the receiver operating characteristic curves of renin, aldosterone, and ARR before washout.

## Results

### Cohort Characteristics

**Figure 1.**
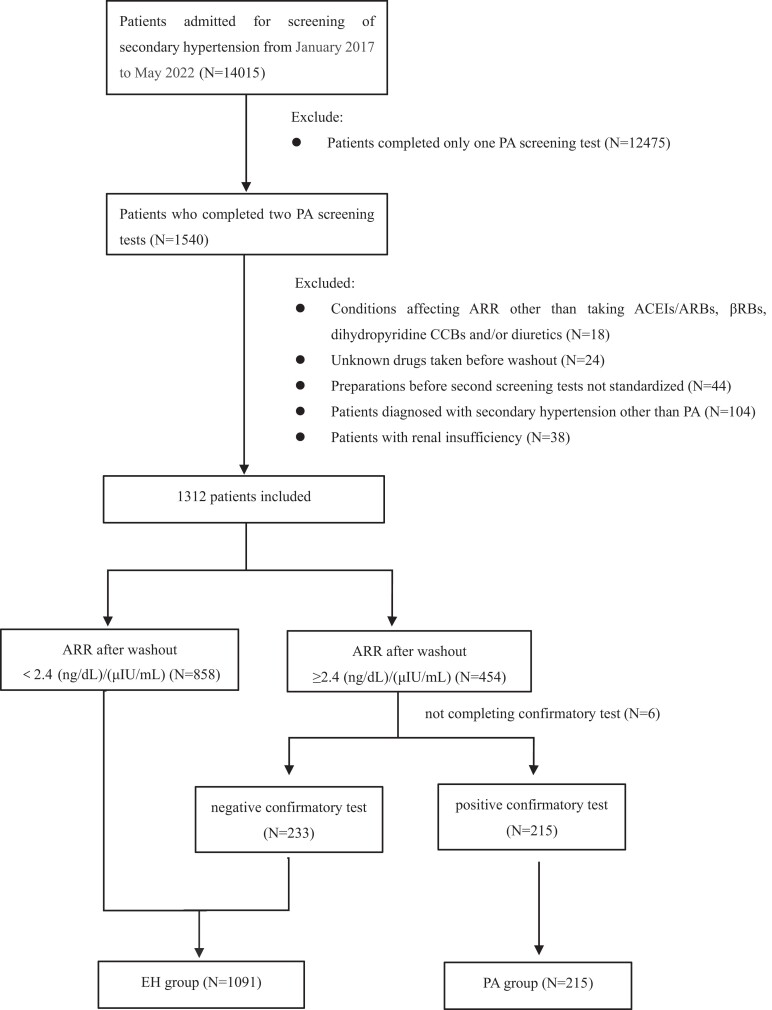
Flowchart of study subjects. Abbreviations: ACEI, angiotensin-converting enzyme inhibitor; ARB, angiotensin-II–blocker; ARR, the aldosterone-to-renin ratio; βRB, β-adrenergic blockers; CCB, calcium channel blocker.

We screened a total of 14 015 patients; 12 709 were excluded, and the final analysis included 1306 patients (836 men and 470 women) with a median age of 50.2 (41.0-59.0) years (Fig. 1). The baseline data was compared between the 1540 patients who completed 2 screening tests and the 12 475 who underwent only 1 screening test [Supplemental Table S1 (20)]. Confirmatory tests showed PA in 215 (16.5%) patients and EH in the remaining 1091 (83.5%) patients. Demographic and baseline characteristics of the patients with PA vs EH are shown in Table 1. In comparison to the EH group, the PA group had older age, higher rate of uncontrolled hypertension (systolic blood pressure ≥ 140 mmHg and/or diastolic blood pressure ≥ 90 mmHg despite treatment with antihypertensives), higher rate of hypokalemia (serum potassium <3.5 mmol/L), higher rate of elevated urinary albumin/creatinine ratio (≥ 30 mg/g), and higher rate of ventricular septal hypertrophy (interventricular septal thickness at diastole ≥12 mm) (all *P* < .05). The number of antihypertensive drugs was also higher in the PA group (median of 3 vs 2 in the EH group; *P* < .001).

**Table 1.** Demographic and baseline characteristics of the patients

Variable	All patients (n = 1306)	Essential hypertension (n = 1091)	Primary aldosteronism (n = 215)	*P* Values
Age (years)	50.2 (41.0-59.0)	49.2 (40.0-58.0)	55.4 (47.0-62.9)	**< .001**
Male sex	837 (64.0)	127 (58.8)	710 (65.1)	.08
BMI (kg/m^2^)	26.7 (24.6-29.4)	26.7 (24.6-29.4)	26.8 (24.6-29.4)	.52
SBP (mmHg)	141 (131-155)	140 (131-155)	144 (132-157)	.18
DBP (mmHg)	85 (77-95)	85 (77-95)	86 (77-94)	.64
SBP ≥ 140mmHg and/or DBP ≥ 90mmHg	810 (62.0)	661 (60.6)	149 (69.3)	.**02**
Heart rate (bpm)	69 (66-73)	69 (66-74)	69 (66-73)	.22
FPG (mmol/L)	5.14 (4.68-5.75)	5.13 (4.66-5.74)	5.19 (4.78-5.80)	.19
Serum K^+^ (mmol/L)	3.84 (3.58-4.10)	3.87 (3.64-4.12)	3.60 (3.30-3.93)	**< .001**
Serum K^+^ <3.5 mmol/L	239 (18.3)	155 (14.2)	84 (39.1)	**< .001**
Serum Na^+^ (mmol/L)	142.5 (140.8-144.1)	142.3 (140.6-143.9)	143.7 (141.8-145.1)	**< .001**
sCr (μmol/L)	80.7 (70.0-92.1)	80.8 (70.0-92.0)	79.4 (68.9-94.4)	.80
ALT (U/L)	22.0 (15.0-33.0)	22.0 (15.0-33.0)	21.5 (15.0-29.0)	.09
UACR (mg/g)*^a^*	12.96 (7.35-26.29)	12.15 (7.20-24.07)	16.76 (8.99-35.69)	**< .001**
UACR ≥ 30mg/g*^a^*	276 (21.3)	210 (19.4)	66 (30.7)	**< .001**
IVSd (mm)*^b^*	10.0 (9.0-11.0)	10.0 (9.0-11.0)	10.0 (9.0-12.0)	**< .001**
IVSd ≥12 mm*^b^*	250 (19.2)	195 (17.9)	55 (25.6)	.**01**
LVPWd (mm)*^b^*	9.0 (9.0-10.0)	9.0 (9.0-10.0)	10.0 (9.0-10.0)	.15
LVPWd ≥12 mm*^b^*	97 (7.4)	81 (7.4)	16 (7.4)	> .99
No. of antihypertensive drugs	2.0 (1.0-3.0)	2.0 (1.0-3.0)	3.0 (2.0-3.0)	**< .001**
1	345 (26.42)	312 (28.6)	33 (15.35)	
2	493 (37.75)	420 (38.5)	73 (33.95)	
3	336 (25.73)	262 (24.01)	74 (34.42)	
4	132 (10.11)	97 (8.89)	35 (16.28)	
ACEI/ARBs	961 (73.5)	788 (72.2)	173 (80.1)	.**02**
β-blockers	535 (40.9)	418 (38.3)	117 (54.2)	**< .001**
CCBs	981 (75.1)	798 (73.1)	183 (84.7)	**< .001**
Diuretics	392 (30.0)	322 (29.5)	70 (32.4)	.40

Abbreviations: ACEI, angiotensin-converting enzyme inhibitor; ALT, alanine transaminase; ARB, angiotensin-II–blocker; BMI, body mass index; CCB, calcium channel blocker; DBP, diastolic blood pressure; FPG, fasting plasma glucose; IVSd, interventricular septal thickness at diastole; LVPWd, thickness of left ventricular posterior wall at diastole; SBP, systolic blood pressure; sCr, serum creatinine; serum K^+^, concentration of potassium in serum; serum Na^+^, concentration of sodium in serum; UACR, urinary albumin/creatinine ratio.

Data are expressed as n (%) or median (25th-75th percentile).

^*a*^n (all patients) = 1295, n (essential hypertension) = 1080, n (primary aldosteronism) = 215.

^*b*^n (all patients) = 1303, n (essential hypertension) = 1088, n (primary aldosteronism) = 215.

### Test Results

**Figure 2.**
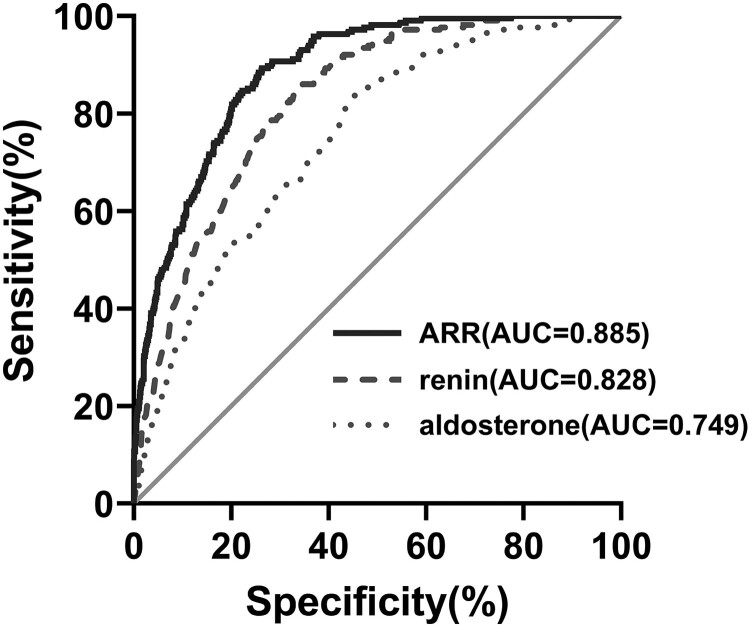
Receiver operating characteristic curve analysis for the plasma aldosterone concentration, renin concentration, and the aldosterone-to-renin ratio for detecting primary aldosteronism.

In comparison to the second screening test, the first screening test yielded lower plasma aldosterone and higher renin and consequently lower ARR in both the PA and EH groups (all *P* < .001, Table 2). The area under the receiver operating characteristic curve for using the first screening test to predict PA was .749 (95% CI .724-.772) with plasma aldosterone, .828 (95% CI .806-.848) with plasma renin, and .885 (95% CI .866-.902) with ARR (Fig. 2). The area under the curve of ARR in the first screening test was significantly higher than aldosterone and renin before washout (both *P* < .001).

**Table 2.** Plasma aldosterone concentration, renin concentration, and ARR prior to and after washout

	Essential hypertension			Primary aldosteronism		
	before washout	after washout	*P*	before washout	after washout	*P*
ARR [(ng/dL)/(μIU/mL)]	.46 (.20-1.23)	1.00 (.50-2.06)	**< .001**	4.51 (1.85-12.41)	8.74 (5.01-21.60)	**< .001**
Renin concentration (μIU/mL)	25.60 (9.30-63.40)	12.90 (5.40-27.60)	**< .001**	3.65 (1.50-9.63)	2.50 (1.00-4.38)	**< .001**
Aldosterone concentration (ng/dL)	11.60 (8.30-16.20)	12.80 (9.20-17.10)	**< .001**	18.55 (13.30-24.03)	20.30 (16.18-27.98)	**< .001**

Abbreviations: ARR, aldosterone-to-renin ratio.

Data are expressed as median (25th-75th percentile).

At a .7 cutoff, ARR in the first screening test had 96.3% (95% CI 94.7-99.2%) sensitivity and 61.2% (95% CI 58.3-64.1%) specificity (Table 3). The PPV and NPV were .33 and .99, respectively. At a lower cutoff of .5, ARR in the first screening test produced slightly higher sensitivity [97.7% (95% CI 94.7-99.2%)] but had much lower specificity [52.0% (95% CI 49.0-55.0%)]. The PPV and NPV were .29 and .99, respectively. At a higher cutoff of 1.0, ARR in the first screening test had much lower sensitivity [90.7% (95% CI 86.0-94.2%)] despite higher specificity [70.3% (95% CI 67.5-73.0%)]. The PPV and NPV were .38 and .97, respectively.

**Table 3.** Diagnostic accuracy of the ARR, renin concentration, and plasma aldosterone concentration before washout at different cutoffs

Cutoffs	TP (N)	FP (N)	FN (N)	TN (N)	Youden index	Sensitivity (95%CI)	Specificity (95%CI)	PPV	NPV
ARR [(ng/dL)/(μIU/mL)]									
>.5	210	524	5	567	.496	97.7 (94.7-99.2)	52.0 (49.0-55.0)	.29	.99
>.6	207	459	8	632	.542	96.3 (92.8-98.4)	57.9 (54.9-60.9)	.31	.99
>.7	207	423	8	668	.575	96.3 (94.7-99.2)	61.2 (58.3-64.1)	.33	.99
>.8	200	384	15	707	.578	93.0 (88.8-96.0)	64.8 (61.9-67.6)	.34	.98
>.9	195	355	20	736	.582	90.7 (86.0-94.2)	67.5 (64.6-70.2)	.35	.97
>1.0	195	324	20	767	.610	90.7 (86.0-94.2)	70.3 (67.5-73.0)	.38	.97
>1.1	193	302	22	789	.621	89.8 (84.9-93.5)	72.3 (69.6-75.0)	.39	.97
>1.2	190	280	25	811	.627	88.4 (83.3-92.3)	74.3 (71.6-76.9)	.4	.97
Renin concentration (μIU/mL)									
≤17.5	194	437	21	654	.502	90.2 (85.5-93.9)	59.9 (57.0-62.9)	.31	.97
≤27.5	205	571	10	520	.430	95.3 (91.6-97.7)	47.7 (44.7-50.7)	.26	.98
≤40	210	684	5	407	.350	97.7 (94.7-99.2)	37.3 (34.4-40.3)	.23	.99
Aldosterone concentration (ng/dL)									
>8	210	835	5	256	.211	97.7 (94.7-99.2)	23.5 (21.0-26.1)	.20	.98
>9	205	766	10	325	.251	95.3 (91.6-97.7)	29.8 (27.1-32.6)	.21	.97
>10.5	192	622	23	469	.323	89.3 (84.4-93.1)	43.0 (40.0-46.0)	.24	.95

Abbreviations: ARR, aldosterone-to-renin ratio; FN, false negative; FP, false positive; NPV, negative predictive value; PPV, positive predictive value; TN, true negative; TP, true positive.

Subgroup analysis was performed in patients who used antihypertensive drugs consisting of β-blockers and those not consisting of β-blockers before washout [Supplemental Table S2 and S3 (20)]. At a .7 cutoff, ARR before washout had 98.3% (95% CI 94.0-99.8%) sensitivity and 58.9% (95% CI 54.0-63.6%) specificity in patients who used antihypertensive drugs consisting of β-blockers. The sensitivity and specificity were 93.9% (95% CI 87.1-97.7%) and 62.4% (95% CI 58.6-66.1%) in patients using antihypertensive drugs not consisting of β-blockers.

Performance of plasma renin and aldosterone is also shown in Table 3. At a cutoff of 27.5μIU/mL, the sensitivity, specificity, PPV, and NPV of renin concentration in the first screening test were 95.3% (95% CI 91.6-97.7%), 47.7% (95% CI 44.7-50.7%), .26, and .98, respectively. The sensitivity, specificity, PPV, and NPV of aldosterone in the first screening test were 95.3% (95% CI 91.6-97.7%), 29.8% (95% CI 27.1-32.6%),.21, and .97, respectively, using 9.0 ng/dL as the cutoff.

## Discussion

In the current study, ARR at a .7 cutoff prior to washout of hypertensive drugs had very high sensitivity (96.3%) and reasonable specificity (61.2%). The PPV and NPV were .33 and .99, respectively. These results encourage the use of ARR without washout as a screening tool for PA.

PA is a frequent cause of secondary hypertension and is associated with higher risk of target organ damage. Previous studies have demonstrated that appropriate treatment of PA resulted in a marked reduction of cardiovascular and renal complications (21-23). Dr. Young at Mayo Clinic suggested that all patients with hypertension be recommended for case detection testing for PA at least once (24). The Japanese Endocrine Society also recommends primary aldosteronism screening by general practitioners in all patients diagnosed with hypertension (7).

Despite increasing awareness, PA remains largely overlooked in daily practice settings (25, 26), due to a variety of practical issues. The reported screening rate is only .1% to 2.1% in patients with features strongly suggestive of PA (11, 26-28). Our findings are beneficial to reduce the time, cost, and risk of altering antihypertensive medications to facilitate “clean” testing in patients detecting for PA. This would greatly improve the screening rate. The proportion of PA in the overall cohort was 16.5% in the current study. Such a rate is consistent with that reported in specialized hypertension clinics (29). The PPV and NPV therefore are applicable to the patient population in specialized centers and perhaps not in primary care settings. Consistent with previous studies (10), plasma aldosterone, renin, and ARR differed significantly prior to vs after washout of antihypertensive drugs. The area under the receiver operating characteristic curve for using the first screening test to predict PA was considerably higher for ARR than either plasma aldosterone or renin. Subsequent analysis therefore focused on ARR.

We examined the diagnostic performance of ARR in the first screening test. The sensitivity in identifying PA was <95% with a cutoff value of .8 or higher and 96.3% when using .7 as the cutoff. For case detection that requires a high sensitivity, a cutoff value of .8 or higher is not satisfactory. On the other hand, lowering the cutoff value to below .7 resulted in only marginal improvement in sensitivity but significant loss of specificity. We therefore believe .7 is the most appropriate cutoff for ARR prior to antihypertensive washout as a screening tool. Further analysis showed that missed diagnosis of PA occurred in 8 subjects with ARR before washout ≤ .7(ng/dL)/(μIU/mL), and they all had unilateral adrenal mass on computed tomography and/or hypokalemia [Supplemental Table S4 (20)]. Besides, subgroup analysis showed that the sensitivity was higher in patients using antihypertensive drugs consisting of β-blockers than those not consisting of β-blockers (98.3% vs 93.9%). This is in accordance with the concept that β-blockers could lead to a false-positive result of ARR.

Given the high prevalence of PA, several approaches have been used to negate the need to discontinue antihypertensive drugs prior to the ARR screening test and simplify the process of diagnosis. A commonly used method is to interpret ARR based on the known effects of antihypertensive medication on the RAAS (8, 10). But this method has several limitations. First, many patients take drugs associated with both false-positive and false-negative ARR, and data interpretation in such patients is difficult. Second, patients with a negative ARR while taking antihypertensive drugs associated with false-negative ARR must undergo a second test after discontinuing antihypertensive drugs.

Another approach to simplify the screening procedure is implementation of PA screening at the time of initial diagnosis of hypertension and prior to any prescription of antihypertensive drugs. This approach is attractive considering the fact that up to 14% of patients with newly diagnosed hypertension have PA (30-33), but it is clearly not pragmatic in the real world due to the vast occurrence of incident hypertension each year and limited facilities that routinely offer ARR testing.

Pilz et al reported 100% sensitivity and 91.8% specificity in detecting PA when using ARR with a 4.33 (ng/dL)/(μIU/mL) cutoff without discontinuing first-line antihypertensive drugs (34). The diagnosis of PA in their study, however, was not based on the standard procedure of ARR screening after washout followed by confirmatory tests. Gallay et al conducted a study in 90 patients without discontinuing antihypertensive drugs and found adrenal adenoma or hyperplasia in 15 patients (17%) with ARR >100 (ng/dL)/(ng/mL/h) [corresponding to 12.2 (ng/dL)/(μIU/mL) if a conversion factor of 8.2 is used, as suggested by the Endocrine Society guideline] (9, 35). But they did not conduct further investigation in the remaining patients. A study in Japanese patients reported 81% sensitivity and 82% specificity in detecting PA when using ARR ≥69 (ng/dL)/(ng/mL/h) [corresponding to 8.4 (ng/dL)/(μIU/mL) when using a conversion factor of 8.2] without discontinuing antihypertensive drugs (36). However, the sensitivity of this cutoff is not satisfying as a screening test. In that study, 27 patients were diagnosed with PA based on the results of both abdominal computed tomography and adrenal scintigraphy in all 55 patients suspected of PA (36), but this diagnostic procedure is not recommended so far.

The current study has several limitations. First, it was a retrospective analysis conducted at a single center. Second, not all patients during the index period underwent 2 screening ARR tests. Third, the European Society of Hypertension suggested that a lower cutoff (between 1.12 and 2.7) should be adopted with chemiluminescence immunoassays in the 2020 guideline (8, 17, 18, 37). Although the cutoff of ARR used in the current study [≥ 2.4(ng/dL)/(μIU/mL)] was in line with the guidelines at the time, this may lead to misdiagnosis of PA. Fourth, adrenal venous blood sampling was not conducted to determine PA subtypes. Prospectively designed studies with multicenter participation and diagnosis independent of ARR are needed.

In conclusion, ARR determined while hypertensive patients are on medications is a sensitive screening tool for PA. Washout of antihypertensive agents could be omitted and PA diagnosis could be excluded if ARR was ≤ .7(ng/dL)/(μIU/mL) before washout.

## Data Availability

Some or all datasets generated during and/or analyzed during the current study are not publicly available but are available from the corresponding author on reasonable request.

## References

[1] Byrd JB , Turcu AF, Auchus RJ. Primary aldosteronism: practical approach to diagnosis and management. Circulation. 2018;138(8):823‐835. 30359120 10.1161/CIRCULATIONAHA.118.033597 PMC6205759

[2] Wannachalee T , Lieberman L, Turcu AF. High prevalence of autonomous aldosterone production in hypertension: how to identify and treat it. Curr Hypertens Rep. 2022;24(5):123‐132. 35165831 10.1007/s11906-022-01176-7

[3] Ariens J , Horvath AR, Yang J, Choy KW. Performance of the aldosterone-to-renin ratio as a screening test for primary aldosteronism in primary care. Endocrine. 2022;77(1):11‐20. 35622194 10.1007/s12020-022-03084-x PMC9242901

[4] Hung A , Ahmed S, Gupta A, et al Performance of the aldosterone to renin ratio as a screening test for primary aldosteronism. J Clin Endocrinol Metab. 2021;106(8):2423‐2435. 34008000 10.1210/clinem/dgab348

[5] Monticone S , Sconfienza E, D'Ascenzo F, et al Renal damage in primary aldosteronism: a systematic review and meta-analysis. J Hypertens. 2020;38(1):3‐12. 31385870 10.1097/HJH.0000000000002216

[6] Monticone S , D'Ascenzo F, Moretti C, et al Cardiovascular events and target organ damage in primary aldosteronism compared with essential hypertension: a systematic review and meta-analysis. Lancet Diabetes Endocrinol. 2018;6(1):41‐50. 29129575 10.1016/S2213-8587(17)30319-4

[7] Naruse M , Katabami T, Shibata H, et al Japan Endocrine Society clinical practice guideline for the diagnosis and management of primary aldosteronism 2021. Endocr J. 2022;69(4):327‐359. 35418526 10.1507/endocrj.EJ21-0508

[8] Mulatero P , Monticone S, Deinum J, et al Genetics, prevalence, screening and confirmation of primary aldosteronism: a position statement and consensus of the working group on endocrine hypertension of the European Society of hypertension. J Hypertens. 2020;38(10):1919‐1928. 32890264 10.1097/HJH.0000000000002510

[9] Funder JW , Carey RM, Mantero F, et al The management of primary aldosteronism: case detection, diagnosis, and treatment: an endocrine society clinical practice guideline. J Clin Endocrinol Metab. 2016;101(5):1889‐1916. 26934393 10.1210/jc.2015-4061

[10] Mulatero P , Bertello C, Veglio F, Monticone S. Approach to the patient on antihypertensive therapy: screen for primary aldosteronism. J Clin Endocrinol Metab. 2022;107(11):3175‐3181. 35964152 10.1210/clinem/dgac460

[11] Jędrusik P , Symonides B, Lewandowski J, Gaciong Z. The effect of antihypertensive medications on testing for primary aldosteronism. Front Pharmacol. 2021;12:684111. 34054559 10.3389/fphar.2021.684111 PMC8155700

[12] Mulatero P , Rabbia F, Milan A, et al Drug effects on aldosterone/plasma renin activity ratio in primary aldosteronism. Hypertension. 2002;40(6):897‐902. 12468576 10.1161/01.hyp.0000038478.59760.41

[13] Seifarth C , Trenkel S, Schobel H, Hahn EG, Hensen J. Influence of antihypertensive medication on aldosterone and renin concentration in the differential diagnosis of essential hypertension and primary aldosteronism. Clin Endocrinol (Oxf). 2002;57(4):457‐465. 12354127 10.1046/j.1365-2265.2002.01613.x

[14] Bossuyt PM , Reitsma JB, Bruns DE, et al STARD 2015: an updated list of essential items for reporting diagnostic accuracy studies. BMJ. 2015;351:h5527. 26511519 10.1136/bmj.h5527 PMC4623764

[15] Funder JW , Carey RM, Fardella C, et al Case detection, diagnosis, and treatment of patients with primary aldosteronism: an endocrine society clinical practice guideline. J Clin Endocrinol Metab. 2008;93(9):3266‐3281. 18552288 10.1210/jc.2008-0104

[16] Chinese Endocrine Society . Expert consensus on diagnosis and management of primary aldosteronism (2016). Chin J Endocrinol Metal. 2016;32(3):188‐195.

[17] Burrello J , Monticone S, Buffolo F, et al Diagnostic accuracy of aldosterone and renin measurement by chemiluminescent immunoassay and radioimmunoassay in primary aldosteronism. J Hypertens. 2016;34(5):920‐927. 27031933 10.1097/HJH.0000000000000880

[18] Rossi GP , Ceolotto G, Rossitto G, et al Prospective validation of an automated chemiluminescence-based assay of renin and aldosterone for the work-up of arterial hypertension. Clin Chem Lab Med. 2016;54(9):1441‐1450. 26824982 10.1515/cclm-2015-1094

[19] Song Y , Yang S, He W, et al Confirmatory tests for the diagnosis of primary aldosteronism: a prospective diagnostic accuracy study. Hypertension. 2018;71(1):118‐124. 29158354 10.1161/HYPERTENSIONAHA.117.10197

[20] Liu X . Performance of aldosterone-to-renin ratio before washout of antihypertensive drugs in screening of primary aldosteronism. Mendeley Data2024. 2024. doi: 10.17632/hp4vbw9dd6.2 PMC11570383 38381080

[21] Wu VC , Wang SM, Huang KH, et al Long-term mortality and cardiovascular events in patients with unilateral primary aldosteronism after targeted treatments. Eur J Endocrinol. 2021;186(2):195‐205. 34851859 10.1530/EJE-21-0836

[22] Hundemer GL , Curhan GC, Yozamp N, Wang M, Vaidya A. Renal outcomes in medically and surgically treated primary aldosteronism. Hypertension. 2018;72(3):658‐666. 29987110 10.1161/HYPERTENSIONAHA.118.11568 PMC6202119

[23] Hundemer GL , Curhan GC, Yozamp N, Wang M, Vaidya A. Cardiometabolic outcomes and mortality in medically treated primary aldosteronism: a retrospective cohort study. Lancet Diabetes Endocrinol. 2018;6(1):51‐59. 29129576 10.1016/S2213-8587(17)30367-4 PMC5953512

[24] Young WF Jr . Diagnosis and treatment of primary aldosteronism: practical clinical perspectives. J Intern Med. 2019;285(2):126‐148. 30255616 10.1111/joim.12831

[25] Liu YY , King J, Kline GA, et al Outcomes of a specialized clinic on rates of investigation and treatment of primary aldosteronism. JAMA Surg. 2021;156(6):541‐549. 33787826 10.1001/jamasurg.2021.0254 PMC8014194

[26] Cohen JB , Cohen DL, Herman DS, Leppert JT, Byrd JB, Bhalla V. Testing for primary aldosteronism and mineralocorticoid receptor antagonist use among U.S. Veterans: a retrospective cohort study. Ann Intern Med. 2021;174(3):289‐297. 33370170 10.7326/M20-4873 PMC7965294

[27] Hundemer GL , Imsirovic H, Vaidya A, et al Screening rates for primary aldosteronism among individuals with hypertension plus hypokalemia: a population-based retrospective cohort study. Hypertension. 2022;79(1):178‐186. 34657442 10.1161/HYPERTENSIONAHA.121.18118 PMC8664996

[28] Jaffe G , Gray Z, Krishnan G, et al Screening rates for primary aldosteronism in resistant hypertension: a cohort study. Hypertension. 2020;75(3):650‐659. 32008436 10.1161/HYPERTENSIONAHA.119.14359

[29] Brown JM , Siddiqui M, Calhoun DA, et al The unrecognized prevalence of primary aldosteronism: a cross-sectional study. Ann Intern Med. 2020;173(1):10‐20. 32449886 10.7326/M20-0065 PMC7459427

[30] Monticone S , Burrello J, Tizzani D, et al Prevalence and clinical manifestations of primary aldosteronism encountered in primary care practice. J Am Coll Cardiol. 2017;69(14):1811‐1820. 28385310 10.1016/j.jacc.2017.01.052

[31] Libianto R , Russell GM, Stowasser M, et al Detecting primary aldosteronism in Australian primary care: a prospective study. Med J Aust. 2022;216(8):408‐412. 35218017 10.5694/mja2.51438

[32] Chen Y , Xu T, Xu J, et al Strain imaging for the early detection of cardiac remodeling and dysfunction in primary aldosteronism. Diagnostics (Basel). 2022;12(2):543. 35204632 10.3390/diagnostics12020543 PMC8871189

[33] Turcu AF , Yang J, Vaidya A. Primary aldosteronism—a multidimensional syndrome. Nat Rev Endocrinol. 2022;18(11):665‐682. 36045149 10.1038/s41574-022-00730-2

[34] Pilz S , Keppel MH, Trummer C, et al Diagnostic accuracy of the aldosterone-to-active renin ratio for detecting primary aldosteronism. J Endocr Soc. 2019;3(9):1748‐1758. 31528833 10.1210/js.2019-00145 PMC6735732

[35] Gallay BJ , Ahmad S, Xu L, Toivola B, Davidson RC. Screening for primary aldosteronism without discontinuing hypertensive medications: plasma aldosterone-renin ratio. Am J Kidney Dis. 2001;37(4):699‐705. 11273868 10.1016/s0272-6386(01)80117-7

[36] Niizuma S , Nakahama H, Kamide K, et al The cutoff value of aldosterone-to-renin ratio for the diagnosis of primary aldosteronism in patients taking antihypertensive medicine. Clin Exp Hypertens. 2008;30(7):640‐647. 18855267 10.1080/10641960802443282

[37] Manolopoulou J , Fischer E, Dietz A, et al Clinical validation for the aldosterone-to-renin ratio and aldosterone suppression testing using simultaneous fully automated chemiluminescence immunoassays. J Hypertens. 2015;33(12):2500‐2511. 26372319 10.1097/HJH.0000000000000727

